# Genetic Instability and Chromatin Remodeling in Spermatids

**DOI:** 10.3390/genes10010040

**Published:** 2019-01-14

**Authors:** Tiphanie Cavé, Rebecka Desmarais, Chloé Lacombe-Burgoyne, Guylain Boissonneault

**Affiliations:** Department of Biochemistry, Faculty of Medicine and Health Sciences, Université de Sherbrooke, Sherbrooke, QC J1E4K8, Canada; tiphanie.cave@usherbrooke.ca (T.C.); rebecka.desmarais@usherbrooke.ca (R.D.); chloe.lacombe-burgoyne@usherbrooke.ca (C.L.-B.)

**Keywords:** spermiogenesis, chromatin remodeling, DNA double-strand breaks, genetic instability, mutations

## Abstract

The near complete replacement of somatic chromatin in spermatids is, perhaps, the most striking nuclear event known to the eukaryotic domain. The process is far from being fully understood, but research has nevertheless unraveled its complexity as an expression of histone variants and post-translational modifications that must be finely orchestrated to promote the DNA topological change and compaction provided by the deposition of protamines. That this major transition may not be genetically inert came from early observations that transient DNA strand breaks were detected in situ at chromatin remodeling steps. The potential for genetic instability was later emphasized by our demonstration that a significant number of DNA double-strand breaks (DSBs) are formed and then repaired in the haploid context of spermatids. The detection of DNA breaks by 3′OH end labeling in the whole population of spermatids suggests that a reversible enzymatic process is involved, which differs from canonical apoptosis. We have set the stage for a better characterization of the genetic impact of this transition by showing that post-meiotic DNA fragmentation is conserved from human to yeast, and by providing tools for the initial mapping of the genome-wide DSB distribution in the mouse model. Hence, the molecular mechanism of post-meiotic DSB formation and repair in spermatids may prove to be a significant component of the well-known male mutation bias. Based on our recent observations and a survey of the literature, we propose that the chromatin remodeling in spermatids offers a proper context for the induction of de novo polymorphism and structural variations that can be transmitted to the next generation.

## 1. Introduction

As one can appreciate from this Special Issue, the proper packaging of the male haploid genome involves finely-regulated molecular events, resulting in a near complete replacement of somatic chromatin and the formation of a highly condensed nucleus. Although the final protamine deposition (protamination) yields a genetically and mechanically stable nucleus, it became rather intuitive that the previous chromatin remodeling steps and resulting change in DNA topology [1] entailed potential genetic hazards. Early observations that H4 hyperacetylation occurs in murine spermatids [2,3] provided the first evidence that chromatin most likely undergoes a transient state of increased sensitivity to endonucleases during this process [4]. H4 hyperacetylation also sets the stage for the bromodomain, testis-specific, protein (Brdt)-mediated histone eviction [5,6]. In situ detection of 3′OH ends in mouse and human spermatids confirmed that H4 hyperacetylation is indeed coincidental, or slightly precedes the formation of the DNA strand breaks that were observed in the whole spermatid population [7]. Because of its potentially significant transgenerational impact, establishing the genetic consequences of this structural transition and the formation of transient DNA strand breaks has been the ultimate objective of our investigation over the past 15 years.

## 2. DNA Double-Strand Breaks (DSBs) are Intrinsic to the Differentiation Program of Spermatids

Our initial observation that transient DNA strand breaks were observed in the whole population of mouse and human spermatids ruled out that a canonical apoptotic process was involved, as discussed below. Evidence of a similar surge in DNA strand breakage was also reported in rats [8], drosophila [9], grasshoppers (*Eyprepocnemis plorans*) [10] and in algae (*Chara vulgaris*) [11]. Several methods were used to confirm that the transient DNA strand breakage in spermatids also included a significant proportion of DNA double-strand breaks (DSBs). These included neutral comet assay, pulse-field gel electrophoresis [12], γH2AX labelling [13] and qTUNEL assay, whereby double-strand breaks were specifically labelled in solution following a prior step involving DNA nicks and gap filling [14]. Indirect evidence of a DSB repair response based on γH2AX expression must however be taken with caution, as the latter has also been associated with chromatin alteration [15]. Direct methods were also used by our group to show that transient post-meiotic DSBs also form in the fission yeast, lending strong support to the highly conserved nature of this mechanism [16]. Despite their transient character, the formation of DSBs in the haploid context of spermatids represents a genetic threat, because repair must rely solely on end joining processes, as outlined below [17,18]. Considering the lower DNA repair activity reported in condensing spermatids, potential misprocessing of these DSBs would be expected to further increase genetic instability.

## 3. Potential Mechanism for DSBs Formation and Repair

Chromatin structure is a key determinant of the meiotic DSB landscape [19] and hotspot specification in meiosis, where it has been shown to arise from a combination of factors acting upon histones, including the histone methyltransferase PRDM9 [20,21]. In the mature sperm, protamine affords similar but periodic loop-sized protection against the combined activity of an endogenous nuclease interacting with the nuclear matrix-associated topoisomerase IIB (TOP2B) [22]. Similarly, the dynamic character of the chromatin structure transition in spermatids must dictate the genomic distribution of the DSBs during the differentiation steps, and it stands to reason that DNA strand break hotspots regions should be found, although the endonucleases involved have yet to be identified. The potential involvement of TOP2B in the transient formation of DSB has been inferred from the synchronous detection of TOP2B and the tyrosyl-DNA phosphodiesterase 1 (TDP1), which are known to resolve topoisomerase-mediated DNA damage [23]. Such DNA breaks may have been created from the simple hindrance of the TOP2B catalytic cycle during the nuclear condensation process in elongating spermatids. Using RNA interference, the requirement for TOP2 activity in the post-meiotic DSB formation has been recently demonstrated in the ciliate *Terahymena thermophila* [24]. The extent of DSB formation in elongating spermatids is yet unknown. As previously proposed [25], TOP2B is expected to relax all free supercoils generated from nucleosome eviction, reducing the DNA linking number (Lk) in steps of two per catalytic cycle. Although a great number of DSBs should be generated in this case, the DNA ends remain concealed, and it may not elicit a DNA damage response. This is in sharp contrast to the action of an endonuclease that would relax larger domains of unconstrained supercoils from simple strand breakage due to its inability to relegate DNA ends. However, as suggested above in studies concerning mature sperm, a controlled fragmentation process involving the combined action of TOP2 activity and endonucleases in differentiating spermatids should also be considered. For instance, such an interaction between TOP2A and endonuclease G has been shown to be involved in caspase-independent apoptotic DNA fragmentation, since the mitochondrial endonuclease G is translocated to the nuclei during apoptosis [26,27]. Without leading to cell death, it has been proposed that the apoptotic machinery could be borrowed for various differentiation processes, including spermiogenesis [28,29]. Multiple caspases were found to be expressed in the residual bodies of the Drosophila spermatids to remove the unneeded cytoplasmic content during the process of individualization, but caspases are seemingly kept away from the nucleus [28]. In accordance with an early report from Smith and Haaf [30], our group has found no evidence of complete canonical nuclear apoptosis in the nuclei of mouse spermatids in agreement with the transient character of the DNA strand break formation. Since we observed nuclear translocation of endonuclease G in the nuclei of elongating spermatids (unpublished data), its potential association with topoisomerase in spermatids is currently under investigation, as this could promote a caspase-independent mechanism that would lead to the observed surge in DNA fragmentation. Such a mechanism could be under the control of PARP1 and PARP2, as they were shown to strongly inhibit TOP2B activity of spermatids both in vivo and in vitro [31]. One major hallmark of apoptosis is the resulting mitochondrial outer membrane permeabilization, which releases endonuclease G and apoptosis-inducing factors (AIF) [32,33,34,35]. Importantly, although the expression of pro-apoptotic factors has not yet been reported in spermatids, mitochondria are nevertheless known to undergo major structural changes during spermiogenesis. While part of them move to the growing flagellum, other starts to aggregate, and are eventually eliminated by Sertoli cells via phagocytosis or autolysis [36]. It is therefore possible that mitochondrial endonuclease G is released during the autolytic destruction of the outer membrane during the chromatin-remodeling steps. It is worth nothing that even limited, regulated mitochondrial permeabilization can produce DNA damage and genomic instability, without leading to cell death [37], supporting the concept that mitochondrial damage could entail controlled yet reversible DNA fragmentation.

In recent studies, the reversible character of such genome-scale apoptotic-like DNA fragmentation (reversal of apoptosis) has been observed in many instances upon the withdrawal of inducers [38,39]. Striking examples of this are the recovery from global DNA fragmentation observed in the African midge (*P. Vanderplanki*) larva after extreme dehydration [40], similar to the extremotolerant tardigrade species (*R. varieornatus*) [41]. The latter has been shown to express a newly identified, highly basic DNA binding protein (Dsup). The activity of Dsup has been shown to protect transfected cells against radiation and ROS-induced DNA breaks, which is somewhat reminiscent of the protective effect against UV-induced DNA damage that we previously reported for transition proteins [42]. Thus, such a recovery from massive DNA fragmentation, coined “anastasis”, indicates that related mechanisms may be operating in spermatids for global DNA repair. However, recovery from apoptosis-like processes can promote mutagenesis and even oncogenic transformation [37], often displaying micronuclei and chromosomal abnormalities [39,43].

Taken together, this compelling evidence suggests that the chromatin remodeling in haploid spermatids, which precludes the use of homologous recombination for templated DSB repair, should create genetic instability [18]. End-joining processes for DSB repair that are likely to operate in a haploid context include single-strand annealing (SSA), microhomology-mediated end joining (MMEJ) or canonical nonhomologous end joining (NHEJ). SSA occurs within repeated sequences and is known to be intrinsically mutagenic [44], whereas, in the absence of canonical NHEJ factors, MMEJ can process the resected DNA ends, using as little as 1-2bp homology when stabilized by PARP [45]. Canonical NHEJ can proceed without sequence homology, and results in insertion, deletion or even chromosomal rearrangement [17]. Whereas meiosis may have evolved mechanisms to prevent these error-prone end-joining processes [18,46], haploid spermatids likely cannot avoid such mutagenic repair mechanisms. However, our initial mapping data indicated that the transient post-meiotic DSBs arise preferentially within repeated elements of the genome, which should minimize the genetic threat associated with the DSB formation [12]. It is important that coding sequences be protected from the global DNA fragmentation process, especially because of the general loss in DNA repair capacity that has been observed as the chromatin remodeling in spermatids proceeds to the final steps [47,48,49]. In pathological conditions, further alteration in the repair capacity of spermatids could lead to a persistence of DSBs in spermatozoa. Unrepaired DSBs in sperm could be of a lesser concern, given the reported efficient DNA repair activity of the oocytes [50].

## 4. First Evidence of Genetic Instability in Spermatids

Trinucleotide repeats (TNRs) are the most unstable DNA sequences, and transgenerational expansion beyond a given threshold has been linked to inherited neuromuscular and neurological disorders in offspring [51,52]. Thus, variations in TNR represent an ideal sentinel to monitor genetic instability as a result of faulty DNA repair or chromatin remodeling. Using a transgenic mouse model, the TNR expansion of a CAG repeat within exon 1 of the human *HD* gene was shown to be limited to post-meiotic events in males, and thus does not involve mitotic replication or homologous recombination between chromosomes [53]. Using this mouse model [54], the purification of spermatids into four distinct populations allowed us to further demonstrate that an increased frequency of longer DNA repeat length occurs just following chromatin remodeling, as was observed in mouse step 15–16 spermatids, which is equivalent to the transition between steps 3 and 4 in human spermiogenesis [55]. Interestingly, based on the increased intensity of the individual repeat length, we estimated that approximately 20% of spermatids displayed a shift to a longer repeat length [56]. In vitro experiments suggested that the increase in free superhelical density that must prevail during histone eviction resulted in expansion at a stabilized hairpin [57]. DNA secondary structures are indeed causative factors of expansion [52], and the free supercoils in spermatids offer an ideal context for hairpin extrusion and stabilization of other alternative non-B DNA conformations at repeated elements, including cruciform and left-handed Z-DNA [58,59]. Because non-B DNA structures are preferential substrates for endonucleolytic incisions [60,61], the striking enrichment of DSBs that we observed at repeated elements of the spermatid’s genome may therefore not be surprising. Thus, monitoring TNRs length variation during the chromatin remodeling provided the first experimental evidence that genetic instability is an important feature of differentiating spermatids.

## 5. DNA Fragmentation in Spermatids and the Male Mutation Bias

Over the past few years, next generation sequencing (NGS) of parent–offspring trios has confirmed the clear male bias for the transmission of de novo mutations. Male-biased mutations include single-nucleotide variants, small insertions–deletions (indels), and structural variations [62,63,64,65,66,67]. Not surprisingly, the greater number of replication cycles in spermatogenesis was originally suspected as the leading cause of de novo mutations, because the male-to-female mutation rate ratio correlates with the male-to-female ratio of the number of cell divisions [68].

The relatively recent observation that transient DNA double-strand breaks are part of the differentiation program of spermatids (and the even more recent results on the genome-wide distribution of these breaks) could explain why only a few reports have considered this process in the etiology of the male mutation bias. Several lines of evidence, however, suggest that the formation of DSBs and repair in the haploid context of spermatids are compatible with the recent NGS data. First, the amount of mutations generated during DNA replication is much lower than the polymerase error rate. Hence, the reliability and extent of DNA repair (or DNA repair rate) becomes prominent in the determination of transmittable de novo mutations [69]. Previous reports confirmed the decline in the general repair capacity during spermiogenesis [49], and the limited response to DSBs in elongating spermatids compared to pre-meiotic cells [47,48,70]. Second, our initial screening in mice indicated that a large part of the transient DSBs in spermatids map to repeated elements of the genome, in accordance with the relative abundance of these intergenic regions. Interestingly, however, DSBs arise at a greater frequency in LINEs and microsatellites relative to their normal representation in the genome [12]. Coincidentally, a higher frequency of de novo mutations was found to arise within repetitive DNA sequences [67], and a strong paternal bias has been reported for mutations within microsatellite repeats [71]. Interestingly, we found the density of DSBs to be four times higher in the Y chromosomes than autosomes, which is compatible with the higher mutability reported for the Y chromosome [72]. Third, and as outlined above, DSBs and end-joining repair processes in haploid spermatids are likely to offer a proper context for male-driven rearrangement. De novo indels and structural variations such as retrotransposon insertions and interchromosomal events were shown to arise preferentially in the paternal germline [66,73], and a similar paternal bias has been reported for copy number variation (CNV) [74,75]. Although replication-based mechanisms could still be responsible for these mutational and structural modifications, they can also arise from the formation of DSBs in the haploid context of spermatids. For instance, in haploid cells, CNV may result from NHEJ, but can also be generated by non-allelic homologous recombination (NAHR) [76], since NAHR is produced by the alignment and subsequent crossover between nonallelic DNA sequence repeats sharing a homology. Both mechanisms require the formation of a DSB [77]. Fourth, it was established that these male-driven variations are generally associated with the transmission of neurodevelopmental disorders [62,63,65,74,78]. For instance, 88% of de novo indels arise on the paternal chromosome and are associated with autism spectrum disorders [65]. When considering only the gene subset, our preliminary gene ontology term analysis showed that DSBs in spermatids arise preferentially within synaptic genes, and with high significance [12]. Hence, the transient DSB formation in spermatids deserves more attention as a potentially significant mechanism by which paternal variation may be transmitted with implications for neurodevelopment.

The transient DSB surge in spermatids may be viewed as a serious threat to the genetic integrity of the differentiating spermatids before it is eventually used for fertilization. However, one must consider that the DSBs are detected in the whole spermatids population and represent the full repertoire of potentially unstable loci seen in a pool of several million cells. The whole genome capture of these DSBs provides a map of their distribution in the whole cell population, if they can be detected by being present in much more than a single cell. A strong DSB hotspot leading to a deleterious mutation or structural variation must therefore be present in a significant subset of spermatids in order to increase the chance of these being selected for fertilization. Pathological conditions that would increase global DNA fragmentation in spermatids could lead to a concomitant increase in the frequency of a given hotspot among cells, thus increasing the chance of that allele being transmitted to the offspring. On the other hand, if a mutational DSBs hotspot is present at a much lower frequency, for instance at 0.01% or lower, then the mutated allele would stand a maximum of 10^−4^ chance of being transmitted for each oocyte being fertilized. In such a case, a beneficial or deleterious mutation would be passed on to the next generation over a much longer (or evolutionary) timescale, provided that a hotspot locus was maintained over time in each species. Interestingly, we observed that selected hotspots are shared between two mouse strains (C57BL/6 vs CD1), suggesting that they can be maintained at least over the evolutionary distance between these inbred and outbred strains. A higher frequency (or density) of such weaker hotspots in a chromosome would confer a faster evolutionary global mutability on an evolutionary timescale, as observed for the Y chromosome.

## 6. DSBs in Lower Eukaryotes

The apparent conservation of transient DSBs in spermatids between mammals prompted us to investigate whether post-meiotic DSBs may be conserved through the eukaryotic domain, and also be associated with gamete formation in yeast (sporulation) [79]. Being more similar to metazoans, fission yeast (*S. pombe*) represents a better alternative than budding yeasts (*S. cerevisiae*) [80]. In addition, synchronous meiosis can be achieved with the *S. pombe pat1-114* mutant, in which a temperature-sensitive Pat1 (Ran1) protein kinase inhibits meiosis by negatively regulating an RNA-binding protein that controls entry into the meiotic S phase, Mei2. Synchronous meiosis can therefore be induced in a timely and predictable fashion by shifting nitrogen-starved cultures from permissive (25 °C) to restrictive (34 °C) temperatures [81]. One striking observation is that synchronized *S. pombe* displayed a similar post-meiotic surge in DSBs in the absence of apoptosis, when meiosis is induced in the *pat1-114* mutant, or even in the wild-type *FY435*/*FY436* strain (azygotic meiosis) [16], suggesting that sporulation, much like spermiogenesis, may display a window of genetic instability. Our conclusion is that transient post-meiotic DSBs may be intrinsic to the gamete differentiation program throughout the eukaryotic domain. As outlined above, a major support for this conclusion recently came from the demonstration that DSBs also arise during post-meiotic steps in the ciliate *Tetrahymena thermophila* [24]. These observations point to the discovery of a highly conserved, physiological mechanism that deserves further investigation regarding its genetic impact and evolutionary consequences. Simple eukaryotic models such as yeast offer the possibility of functional genetics analyses, to identify the endonuclease(s) responsible for the transient DSB formation, and eventually determine their impact on adaptation and evolution over several generations. In-gel nuclease assays in the synchronized pat1-114 mutant have already led to the identification of a candidate mitochondrial endonuclease (Pnu1), an homolog of the *S. cerevisiae* Nuc1p that has been described as part of the caspase-independent apoptotic pathway [82]. Interestingly, the mammalian homolog of Nuc1p is the mitochondrial endonuclease G (Endo G) discussed above, which is also involved in caspase-independent apoptosis. These early observations in yeast lend support to the proposal that the transient post-meiotic DSBs may indeed borrow components of the apoptotic machinery in a controlled, reversible manner.

## 7. Conclusions and Future Directions

Given the non-templated DNA repair in haploid spermatids, transient DSB formation may represent an important component of the male mutation bias and the etiology of neurological disorders, adding to the genetic variation provided by meiosis. Repair heterogeneity at these potential hotspots would produce a repertoire of genetic polymorphisms, given the large population of spermatozoa produced over time. In addition to the chromosome reshuffling provided by meiosis, each offspring would also inherit a given set of mutations created by the chromatin remodeling in the spermatid of origin. Because synaptic genes were found to be specifically targeted by DSBs and should therefore harbor more mutational hotspots, pathological conditions (or aging) leading to a global rise in DSB formation would then increase the odds of transmitting de novo variation in neurodevelopmental genes. This hypothesis is thus in agreement with the reported correlation between the father’s age at conception and the risk of transmitting neurological disorders. Further investigation should therefore be aimed at deciphering whether mutational DSBs hotspots arise within neurodevelopmental genes and how they are altered under pathological conditions, or following exposure to xenobiotics. Monitoring the distribution and number of DSBs in elongating spermatids should be clearly emphasized, as they represent a much higher threat compared to single-strand breaks. Despite the reported DNA repair capacity of the oocyte, the transgenerational consequences of an increased number of persistent DSBs in sperm deserves some attention for future investigations.
